# Designing a Natural Experiment to Evaluate a National Health Care–Community Partnership to Prevent Type 2 Diabetes

**DOI:** 10.5888/pcd10.120149

**Published:** 2013-01-31

**Authors:** Ronald T. Ackermann, Ann M. Holmes, Chandan Saha

**Affiliations:** Author Affiliations: Ann M. Holmes, Chandan Saha, Indiana University School of Medicine, Indianapolis, Indiana.

## Abstract

To address the growing incidence of type 2 diabetes in the United States, UnitedHealth Group, the YMCA of the USA, and the Centers for Disease Control and Prevention have partnered to bring a group-based adaptation of the Diabetes Prevention Program lifestyle intervention to a national scale. Researchers at Northwestern and Indiana universities are collaborating with these partners to design a robust evaluation of the reach, effectiveness, and costs of this natural experiment. We will employ a quasi-experimental, cluster-randomized study design and combine administrative, clinical, and programmatic data from existing sources to derive reliable, timely, and policy-relevant estimates of the program’s impact and potential for sustainability. In this context, evaluation results will provide information about the unique role of a health care–community partnership to prevent type 2 diabetes.

## Introduction

An estimated 79 million Americans have prediabetes and are at high risk for developing type 2 diabetes in the next 5 to 10 years (1,2). Intensive population-based efforts are needed to reduce the development of type 2 diabetes, over a short time, among people who have prediabetes (3). To help address this issue, UnitedHealth Group (UHG), the YMCA of the USA (the Y), and the Centers for Disease Control and Prevention (CDC) have partnered to create a low-cost, group-based adaption of the Diabetes Prevention Program’s (DPP’s) lifestyle intervention for implementation on a national scale.

The DPP clinical trial demonstrated the efficacy of a behavior-based lifestyle intervention to prevent or delay more than half of new cases of type 2 diabetes among adults at high risk (4). Because the DPP promotes healthful diet and moderate increases in physical activity to achieve modest weight loss, it also has benefits beyond diabetes prevention, such as improving other cardiovascular risk factors, reducing health care expenditures, and enhancing well-being (5–9). The DPP’s high programmatic costs and the frequency of ongoing face-to-face visits have made it challenging to implement routinely in the real world (10).

Community delivery of adapted versions of the DPP have demonstrated promise for achieving weight losses consistent with the DPP trial for about one-eighth the cost of the original intervention design (11–13). In 2010, UHG partnered with the Y and CDC to develop 1) standards for recognition of community organizations that offer a program consistent with the DPP; 2) new infrastructures for the training of a nonclinical diabetes prevention workforce to deliver such a population-based program; 3) processes targeting employers, health professionals, and high-risk health plan enrollees to identify people with prediabetes in the general population; and 4) initiatives to encourage such high-risk people to enroll in a community-based DPP intervention. UHG and the Y also collaborated to develop a payment structure that encourages maximal attendance and achievement of at least a 5% weight loss goal for each participant. By combining new analytic and outreach procedures with performance-based payments for the DPP, UHG has constructed a novel preventive-health benefit design that aims to expand the reach and cost-effectiveness of the diabetes prevention programming that the Y offers nationally. 

The success of this initiative depends on the efficient identification of high-risk adults in the population and the willingness of those adults to enroll and maintain participation in the program (13). However, the optimal mix of strategies to maximize program participation is unknown, and the potential for financial sustainability of the program depends on whether the health improvements achieved by greater participation in the DPP are associated with reductions in future health care expenditures. Learning whether the costs and benefits of the program are distributed equitably among all high-risk people in the population, regardless of age, race, culture, or economic context, is also important.

UHG, the Y, and researchers at Northwestern and Indiana universities have partnered to design an evaluation of this natural experiment that will be both pragmatic and rigorous. Our aims are to evaluate whether 1) UHG efforts to identify and engage high-risk adults can efficiently promote use of the Y program; 2) participation in this model for DPP delivery results in meaningful weight loss; 3) use of the program reduces the need for medications to treat diabetes, high blood pressure, or high cholesterol; and 4) DPP participants have lower overall health care use and costs.

## Evaluation Design

Our evaluation will focus on the combined elements of CDC workforce development, UHG engagement activities, and a Y model for DPP delivery that involve performance-based payments from UHG to maximize DPP participation and weight-loss effectiveness. This natural experiment will include more than 10,000 DPP participants in approximately 500 community-based DPP program sites in 44 cities throughout the United States.

### Data sources and outcome metrics

Data from existing administrative, clinical, and Y sources will be evaluated. A dedicated electronic tracking and billing database, developed by UHG to help the Y administer the DPP, will allow us to analyze attendance and weight loss for program participants. Medical, pharmacy, and laboratory claims, available for all UHG enrollees, will allow us to compare changes in total and sector-specific (eg, inpatient) health care expenditures among different groups of enrollees regardless of DPP participation. Pharmacy claims will enable us to assess changes in treatment intensity for conditions linked to obesity and high metabolic risk (high blood pressure, high cholesterol, diabetes) (14). For subgroups of UHG enrollees with available test results associated with laboratory claims (approximately 20% of claims submitted nationally), we will evaluate changes in total cholesterol, low-density lipoprotein cholesterol, and hemoglobin A1c levels. Finally, data provided by the Y will enable us to explore whether regional variation in the structure and process of program implementation can help explain geographic differences in key program outcomes.

### Sampling and analysis plan

One strategy used by UHG to identify prediabetes and to enroll high-risk adults in a Y-based DPP intervention involves large-scale blood glucose or hemoglobin A1c testing as part of a workplace wellness or risk assessment initiative. Because these initiatives are deployed at an employer level, we have designed a cluster-randomized encouragement trial (CRET) as an innovative component of our research design (15,16). The CRET design will allow for comparisons with a randomized control group without interfering with the natural implementation of the program.

In select regions, UHG will provide the research team with a list of large employers whom it would aim to engage prospectively for on-site testing. We will randomly assign these employers to sequential waves of outreach and release them back to UHG to initiate encouragement procedures at a rate that matches the capacity of the UHG outreach team. As some employers are released into the active encouragement arm, others will remain in abeyance to serve as controls until being released at a future date (Figure). By using an intent-to-treat framework, clients who are actively encouraged (and far more likely to participate in the DPP) can be compared with clients who are not encouraged until a later time (17).

**Figure:**
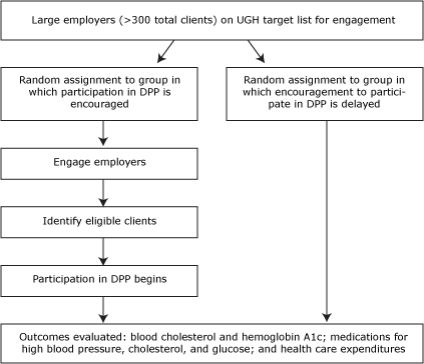
Cluster randomized encouragement design to be used in the Diabetes Prevention Program (DPP) by UnitedHealth Group (UHG).

Although people cannot be randomized to attending the DPP, the CRET design will also enable us to analyze the treatment response among those who do elect to participate (18). Typically, comparisons of such self-selected subgroups can introduce selection bias. However, through the use of instrumental variables analysis (18–21), the CRET design will enable us to use the status of random assignment (ie, encouragement now vs encouragement later) as an instrumental variable to construct a robust estimate of the treatment effect of the program while minimizing the threat of selection bias.

The evaluation will focus on comparable samples of high-risk UHG clients who have prior claims-based evidence of diagnosed prediabetes (*International Classification of Diseases, Ninth Revision, Clinical Modification* [ICD-9-CM] code 790.21 or 790.22) or other indications of high metabolic risk (eg, diagnosed metabolic syndrome [ICD-9-CM code 277.7] or multiple metabolic traits such as overweight and obesity; high blood pressure; abnormal blood cholesterol). In this sample, the study will compare differences between randomized groups in the changes in laboratory tests for blood cholesterol and hemoglobin A1c, medication treatment intensities, and patterns and overall expenditures for health care. Total per-person-per-month (PPPM) health care expenditures will be compared by using 24 months of baseline and 12 month of follow-up data. We expect the cluster-randomization to yield a sample of approximately 60 employers and a minimum of 12,500 high-risk employees in each arm. Under reasonable assumptions that fewer than 30% of the observations may be missing (ie, as clients withdraw from the health plan) and that the intracluster correlation coefficient (ie, within employer) will be no more than 3%, we should have more than 80% power to detect mean differences in total health care costs as small as $300 during the 12 months of follow-up.

One possible challenge of using a CRET design is that it may prove more powerful for evaluating the effects of UHG’s encouragement efforts than for evaluating direct effects of exposure to the DPP. If, for example, the number of employers randomized to the “active encouragement” arm exceeds the capacity of UHG to successfully engage them to identify high-risk employees, then the overall “dose” of DPP exposure in the active arm will be lower than anticipated, and statistical power of the study could be reduced.

## Conclusion

By using strong quasi-experimental methods mapped to the naturally occurring rollout plans of UHG and the Y, we aim to implement a robust study of this adaptation of the DPP program and to guide future policies about its role in the ongoing national fight against type 2 diabetes. In addition to informing UHG and the Y, our evaluation will provide information about how CDC and other public health and policy stakeholders can leverage natural experiments to build the knowledge base necessary for identifying effective policies to battle this and other population health challenges.
